# Tea leaf exosome-like nanoparticles (TELNs) improve oleic acid-induced lipid metabolism by regulating miRNAs in HepG-2 cells

**DOI:** 10.1186/s40643-025-00844-1

**Published:** 2025-02-10

**Authors:** Xuanhao Lei, Haonan Li, Sibei Chen, Bing Li, Huili Xia, Jun Li, Feng Guan, Jian Ge

**Affiliations:** 1 College of Life Sciences, China Jiliang University, 258 XueYuan Street, XiaSha Higher Education Zone, Hangzhou, 310018 Zhejiang Province People’s Republic of China; 2 Taizhou Food and Drug Inspection and Research Institute, Taizhou, 318000 Zhejiang Province People’s Republic of China

**Keywords:** Nanoparticles, Preparation and characterization, Lipid metabolism, miRNA modulation, Tea

## Abstract

Tea is a widely consumed beverage globally, but the tea industry faces a significant waste management challenge. In this study, we developed tea leaf exosome-like nanoparticles (TELNs) with an average size of 274 ± 24.7 nm and a zeta potential of -20.6 ± 0.78 mV, using polyethylene glycol (PEG) 6000 precipitation followed by ultracentrifugation. Structural analysis confirmed that TELNs are composed of lipids, proteins, and RNAs. In vitro assays on HepG-2 cells revealed that TELNs are non-toxic at concentrations up to 300 µg/mL and can be efficiently internalized. TELNs exhibited significant antioxidant capacity and were able to significantly ameliorate H_2_O_2_-induced oxidative stress, increase the viability and reduce the accumulation of ROS in Hepg-2 cells. Notably, TELNs significantly alleviated OA-induced lipid metabolic disorders and hepatocellular injury. Further molecular analysis revealed that TELNs downregulated the expression of miR-21-5p, miR-17-3p, and miR-107, leading to the upregulation of their target genes PPARα, CYP7A1, and CPT-1A, which contributed to the improvement of lipid metabolism. This study is the first to demonstrate the lipid metabolism regulation potential of TELNs, providing new insights into their underlying mechanisms and helping to develop new therapeutic strategies for lipid metabolism-related diseases. Furthermore, it expands the scope of tea use and helps to reuse tea residues.

**Graphical Abstract:**

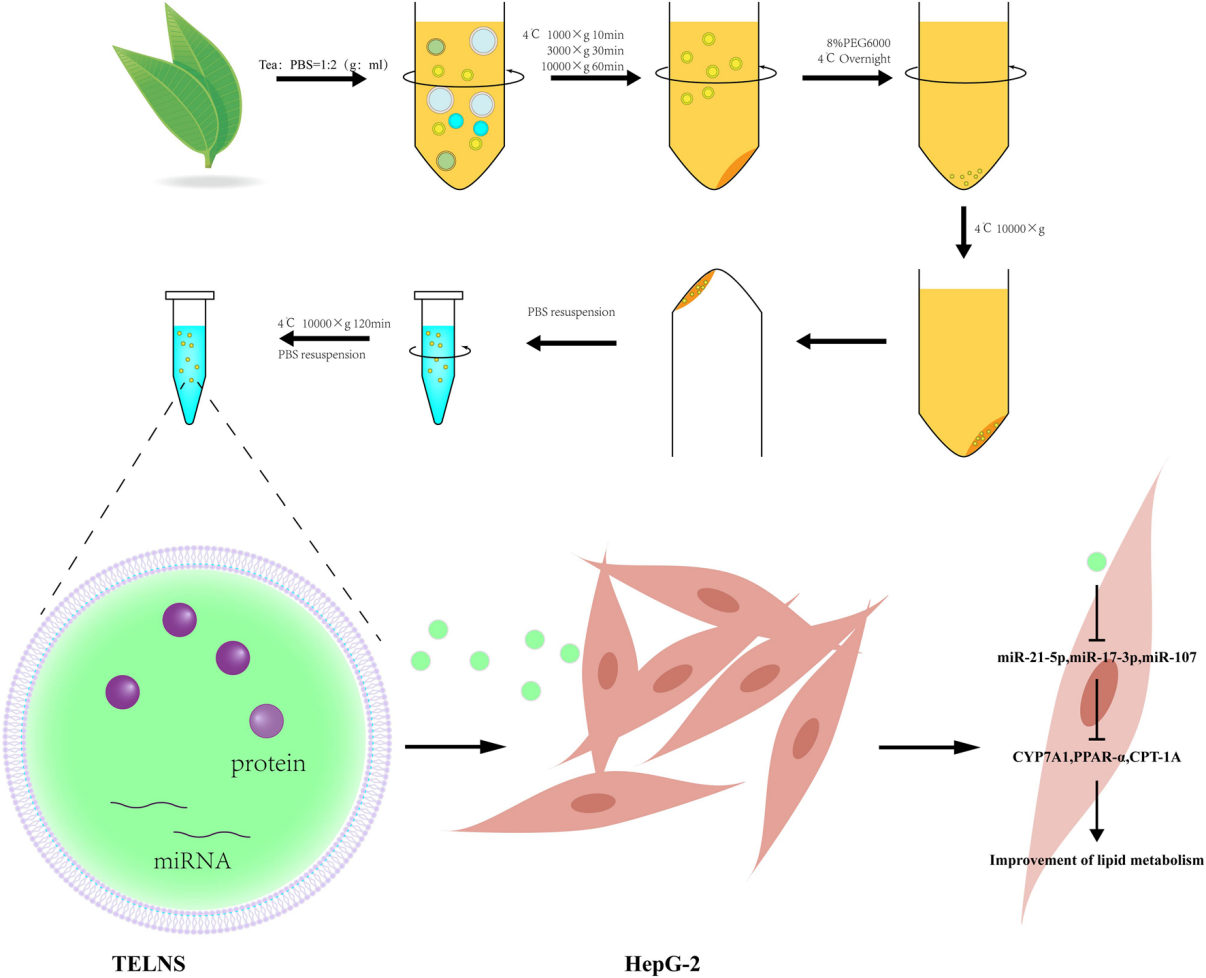

**Supplementary Information:**

The online version contains supplementary material available at 10.1186/s40643-025-00844-1.

## Introduction

Tea is a globally popular beverage, and in China, its medicinal properties are valued alongside its flavor (Pan et al. 2022). The ancient text *Shennong Bencao Jing* states, "Tea is bitter in taste; consuming it enhances cognition, reduces the need for sleep, lightens the body, and sharpens vision." Modern research has identified various bioactive compounds in tea leaves, such as polyphenols, polysaccharides, flavonoids, and amino acids, which have demonstrated antioxidant, antitumor, anti-inflammatory, and lipid-lowering effects (Zhou et al. 2019; Talib et al. 2024; Winiarska-Mieczan et al. 2021). However, due to the taste, people commonly use spring tea leaves, while summer and autumn tea leaves are discarded, which leads to a great waste of resources (Xiao et al. 2021). Therefore, the utilization and development of tea resources are important issues in the current industry.

Plant-derived exosome-like nanoparticles (PELNs) are vesicles extracted and purified from plants, ranging in diameter from 50 to 1000 nm (Pinedo et al. 2021). These nanoparticles exhibit a comparable composition and structural characteristics with mammalian exosomes, including a lipid bilayer membrane and encapsulated components such as RNA and proteins (Feng et al. 2023). Due to their advantages—such as low toxicity, structural stability, ease of absorption and internalization by mammalian cells, and suitability for large-scale production (Bai et al. 2024)—PELNs have gained significant attention in drug delivery (Dad et al. 2021) and therapeutic applications (Kim et al. 2022). Ultracentrifugation is currently regarded as the "gold standard" for PELN extraction and purification, and is widely used to successfully isolate PELNs from plant sources, including strawberries (Perut et al. 2021), ginseng (Seo et al. 2023), ginger (Zhang et al. 2016b), and apples (Trentini et al. 2022), facilitating further research. Additionally, polyethylene glycol (PEG) precipitation is favored for its simplicity, safety, and scalability (Zhao et al. 2024). This method has been employed in the extraction of PELNs from garlic (Liu et al. 2023), ginger (Kalarikkal et al. 2020) and Arabidopsis (Jokhio et al. 2024), with subsequent characterization studies being conducted. Research demonstrates that PELNs hold significant potential in cancer therapy (Yi et al. 2024), lipid metabolism regulation (Pang et al. 2024), and the mitigation of intestinal inflammation (Yin et al. 2022), making the investigation of PELNs from various foods and medicinal plants a prominent area of current research.

In order to improve the utilization of tea resources, further study of the active ingredients of tea is necessary to expand its applications. Recently, attention has turned to PELNs from tea leaves (TELNs). For instance, Chen et al. (2023) reported that TELNs inhibit breast cancer progression by inducing apoptosis. Similarly, Zu et al. found that oral administration of TELNs effectively suppresses inflammatory bowel responses, helps restore damaged colonic barriers, and promotes the diversity and overall richness of gut microbiota, thereby contributing to the prevention or alleviation of inflammatory bowel disease and colitis-associated colon cancer (Zu et al. 2021). Cho et al. demonstrated that TELNs outperform tea extracts in preventing skin aging (Cho et al. 2022), promoting skin regeneration, and maintaining the skin barrier. However, the role and mechanisms of TELNs in regulating lipid metabolism remain unexplored. Given that the biological activities of PELNs often reflect those of their source plants (Kim et al. 2022), we hypothesize that TELNs may also influence lipid metabolism.

In this study, we isolated TELNs from tea leaves using a combination of PEG precipitation and ultracentrifugation techniques. We conducted in vitro experiments to assess the antioxidant capacity, safety, and cellular uptake. Furthermore, we evaluated the regulatory effects of TELNs on lipid metabolism in HepG-2 cells using an oleic acid-induced high-fat model. Our research results demonstrate that TELNs downregulate the expression of miR-21-5p, miR-17-3p, and miR-107 in HepG-2 cells, leading to the upregulation of their target genes, thereby improving OA-induced lipid metabolism disorders and hepatocyte injury.

## Materials and methods

### Extraction and characterization of TELNs

TELNs were extracted using a combined polyethylene glycol (PEG) precipitation and ultracentrifugation approach. Fresh tea leaves (Fuyun 6) were thoroughly washed and then juiced in a juicer at a ratio of 1:2 (g:mL). The juice was then filtered through a sieve to remove solid residues. The filtered juice was then subjected to sequential centrifugation at 1000g, 3000g, and 10000g at 4 °C for 10 min, 30 min, and 60 min, respectively. The resulting supernatant was mixed with an equal volume of a pre-chilled 16% PEG6000 (Rhawn, CN) solution and incubated at 4 °C overnight (> 12 h). Subsequently, the mixture was centrifuged at 10000g at 4 °C for 1 h. The supernatant was discarded, and the centrifuge tubes were inverted on a bench for 5 min to remove excess liquid. The pellet was resuspended in PBS to yield a crude TELNs solution. This crude solution was further purified by centrifugation at 100000g at 4 °C for 2 h (Optima L-100XP, USA). The supernatant was discarded, and the final pellet was resuspended in PBS to obtain the TELNs solution. Protein content was quantified using the BCA protein assay kit (NJJCBIO, CN), and the solution was stored at −80 °C. The sizes of TELNs were determined by dynamic light scattering (DLS, Nano-ZS 90 size analyzer, UK).

The lipids from the TELNs were extracted using the Folch method and analyzed by thin-layer chromatography (Liu et al. 2020). The proteins were denatured by boiling and analyzed by SDS-PAGE electrophoresis with Coomassie Brilliant Blue staining. The RNA was extracted using RNAiso plus (Takara, JP) and analyzed by 1.5% agarose gel electrophoresis, with RNase - and RNase + incubated together for 2 h (Kalarikkal et al. 2020).

### TEM detection

10 μL TELNs were applied to a copper grid and allowed to settle for 1 min, after which excess liquid was absorbed with filter paper. 10 μL uranyl acetate were then added to the copper grid and allowed to settle for 10 min, with excess liquid absorbed by filter paper. The sample was air-dried at room temperature for several minutes before being imaged using a transmission electron microscope (TEM, Hitachi HT7700, JP) at 80 kV and 60,000 × magnification.

### HepG-2 cell culture

Human liver cancer cells HepG-2 were purchased from Chinese Academy of Sciences (Shanghai, CN). The culture medium consisted of 89% Dulbecco's Modified Eagle Medium (DMEM), 10% Fetal Bovine Serum (FBS, Even green, CN), and 1% Penicillin–Streptomycin (Solarbio, CN). Cells were cultured in T25 flasks and incubated at 37°C with 5% CO2 in a carbon dioxide incubator.

The cells were digested with trypsin (Solarbio, CN) and seeded into 96-well plates at a density of 1 × 104 cells per well. After attachment, the OA group was incubated with complete medium containing 0.2 mM oleic acid (OA, Aladdin, CN), while the TELNs groups were incubated with complete medium containing 0.2 mM oleic acid and TELNs at low (L, 100 µg/ml), medium (M, 200 µg/mL), and high (H, 300 µg/mL) concentrations for 24 h. Following the 24-h incubation, the cells were lysed with Triton X-100, and the levels of Triglycerides (TG), Total cholesterol (TC), Low density lipoprotein cholesterol (LDL-C), High density lipoprotein cholesterol (HDL-C), Aspartate aminotransferase (AST) and Alanine aminotransferase (ALT) were measured according to the instructions provided by the respective assay kit (NJJCBIO, CN).

An oxidative damage model of HepG-2 cells was established using complete medium containing 0.7 mM H₂O₂ (Aladdin, CN). The L, M, and H groups were treated with complete medium containing 0.7 mM H₂O₂ and the corresponding concentrations of TELNs (100, 200, 300 µg/mL). After 24 h of co-incubation, cell viability was measured using the CCK-8 assay kit (Solarbio, China).

### Measurements of intracellular ROS levels

HepG-2 cells (2 × 10^4^ cells/well) were cultivated in a 96-well plate and exposed to 0.5 mM H_2_O_2_ in the presence of varying concentrations of TELNs (0.1 mg/mL, 0.2 mg/mL, 0.3 mg/mL) for 24 h. Following the treatment, the cells were thoroughly washed with PBS and incubated with 10 μM DCFH-DA (Beyotime, CN) at room temperature for 30 min. Subsequent to this, the fluorescence intensity was quantitatively analyzed using a fluorescence plate reader, with excitation and emission wavelengths set at 485 nm and 535 nm, respectively.

### In vitro antioxidant assay

The DPPH radical scavenging activity was determined using a DPPH Radical Scavenging Activity Assay Kit (Yuanye, CN). Vitamin C (Vc) served as the positive control at concentrations of 2, 4, 6, 8, 10, 15, and 20 μg/mL. TELNs were diluted with PBS to match these concentrations for comparative analysis.

Hydroxyl radical scavenging activity was evaluated using the salicylic acid method. Vitamin C (Vc) was also used as a positive control at concentrations of 0.1, 0.2, 0.4, 0.6, 0.8, and 1 μg/mL. The concentrations of TELNs tested were 0.4, 0.8, 1, 1.2, 1.6, and 2 μg/mL.

The ABTS radical scavenging activity was measured using an ABTS Radical Scavenging Activity Assay Kit (NJJCBIO, CN). A standard curve was generated using Trolox, and the results were expressed as Trolox equivalents. The concentrations of TELNs tested were 10, 20, 50, 100, and 200 μg/mL.

### Cellular uptake

TELNs were labeled with the fluorophore PKH26 (Umibio, CN), and residual dye was removed by ultracentrifugation. The labeled TELNs were then incubated with HepG-2 cells for 24 h. After incubation, the cells were fixed with 4% paraformaldehyde (PFA, Rhawn, CN) for 10 min and subsequently stained with DAPI (Solarbio, China) to visualize the cell nuclei. Imaging was performed using a confocal laser scanning microscope, with excitation wavelengths set at 340 nm for DAPI and 551 nm for PKH26.

### Oil red O stain

Oil Red O staining was conducted using the Modified Oil Red O Staining Kit (Beyotime, CN). Briefly, cells were cultured in a 24-well plate and divided into three groups: NC (Negative control), OA, and TELNs. The OA group was treated with 0.2 mM oleic acid (OA), while the TELNs group received both 0.2 mM OA and 300 μg/mL TELNs. After 24 h of co-incubation, cells were fixed with 4% paraformaldehyde (PFA) for 10 min. Staining was performed according to the manufacturer’s protocol. Images were captured using an inverted microscope.

### RT-qPCR

Total RNA was extracted from samples using TRIzol® Plus RNA Purification Kit (Thermo Fisher, USA) and RNase-Free DNase Set Kit. Quantitative PCR primers were designed using Primer Premier 6.0 and Beacon designer 7.8 software and then synthesized by Shanghai Sangong Biotechnology Co. The primer sequences are shown in Table 1. RT-qPCR was performed using Power SYBRTM Green PCR Master Mix (Applied Biosystems, Inc., USA) and CFX384 Multi-function Real-Time PCR Instrument (Bio-Rad, USA). 3 replicates were performed for each sample, and the relative expression level of each gene was statistically analyzed by 2^−ΔΔCt^.

**Table 1:** RT-PCR primers

Gene	GenBank accession	Reverse transcription primer sequences (5′–3′)	Size (bp)
PPARα	CR456547.1	CCCTCCTCGGTGACTTATC	111
		GTAATGATAGCCTGAGGCCTTGT	
CYP7A1	NM_000780.4	CCTCCAGTCTCCTCTAACTCA	126
		GTCCCGCCTTGTAAGATCTCT	
CPT1A	NM_001876.4	CACATTCAGGCAGCAAGAGC	121
		CGGAGCAGAGTGGAATCGT	
GAPDH	NM_002046.5	CCATGACAACTTTGGTATCGTGGAA	107
		GGCCATCACGCCACAGTTTC	

### RT-qPCR detection for miRNAs

The miRNA was synthesized by reverse transcription with SuperScript^™^ III reverse transcriptase (Thermo Fisher, USA). Quantitative PCR primers were designed with Primer Premier 6.0 and Beacon designer 7.8 software, and then synthesized by Shanghai Sangong Bioengineering Co. The primer sequences and RT-qPCR conditions are shown in Tables 2 and 3, respectively. RT-qPCR was performed using Power SYBR^®^ Green PCR Master Mix (Applied Biosystems, Inc., USA) and CFX384 Multi-functional Real-Time PCR Instrument (Bio-Rad, USA). each sample was repeated three times, and the relative expression level of each gene was statistically analyzed by 2^−ΔΔCt^.

**Table 2:** Real-time PCR primers of miRNA

Gene	Forward primer and universal primer (5'–3')
hsa-miR-21-5p-RT	GTCGTATCCAGTGCAGGGTCCGAGGTATTCGCACTGGATACGACATAAATTT
hsa-miR-17-3p-RT	GTCGTATCCAGTGCAGGGTCCGAGGTATTCGCACTGGATACGACTCAACA
hsa-miR-107-RT	GTCGTATCCAGTGCAGGGTCCGAGGTATTCGCACTGGATACGACTGATAG
U6-F	GTCGTATCCAGTGCAGGGTCCGAGGTATTCGCACTGGATACGACATAAATTT

**Table 3:** Real-Time PCR conditions of miRNA

Conditions	Reaction systems (20 μL)
Sterile distilled water (SDW)	8.0
Power SYBR^®^ green master mix	10.0
Forward primer (10 μM)	0.5
micro-R (10 μM)	0.5
cDNA	1.0

Initial denaturation at 95 ℃ for 1 min; 40 Cycles (95 ℃ for 15 s, 60 ℃ for 25 s)

### Dual luciferase assay for miRNA-mRNA interaction

The pmirGLO vector containing CYP7A1, PPARα, CPT1A-WT or MUT 3^/^-UTR was constructed according to literature methods, respectively. 293T cells were homogeneously inoculated in 96-well plates and co-transfected when the cell density reached 80%. The miRNA mimic or NC was co-transfected with WT or MUT vector according to the instructions of Lipofectamine TM 3000 transfection reagent (Thermo Fisher, USA). After 48 h of incubation, each well was washed twice with PBS. 250 µL of 1 × PLB lysate was added and cells were lysed at room temperature. The luciferase activity of firefly and Renilla was measured using the Dual Luciferase Reporter Gene Assay System (Promega Co., Ltd, USA) according to the operating instructions. Firefly luciferase activity was then normalized to firefly luciferase activity based on Renilla luciferase activity.

### Data analysis

Data were expressed as mean ± standard deviation (SD). Statistical analysis was performed using SPSS 26.0, and one-way analysis of variance (ANOVA) was used to analyze the level of significance of the data between groups. The level of significance was set at p < 0.05(*), p < 0.01(**), p < 0.001(***) and p < 0.0001(****) for all statistical analysis.

## Results and discussion

### Extraction and characterization of TELNs

TELNs were enriched using polyethylene glycol (PEG) during the pretreatment stage. Following the combination of the enriched crude solutions from multiple rounds, the samples were subjected to ultracentrifugation for further purification.

**Fig. 1:**
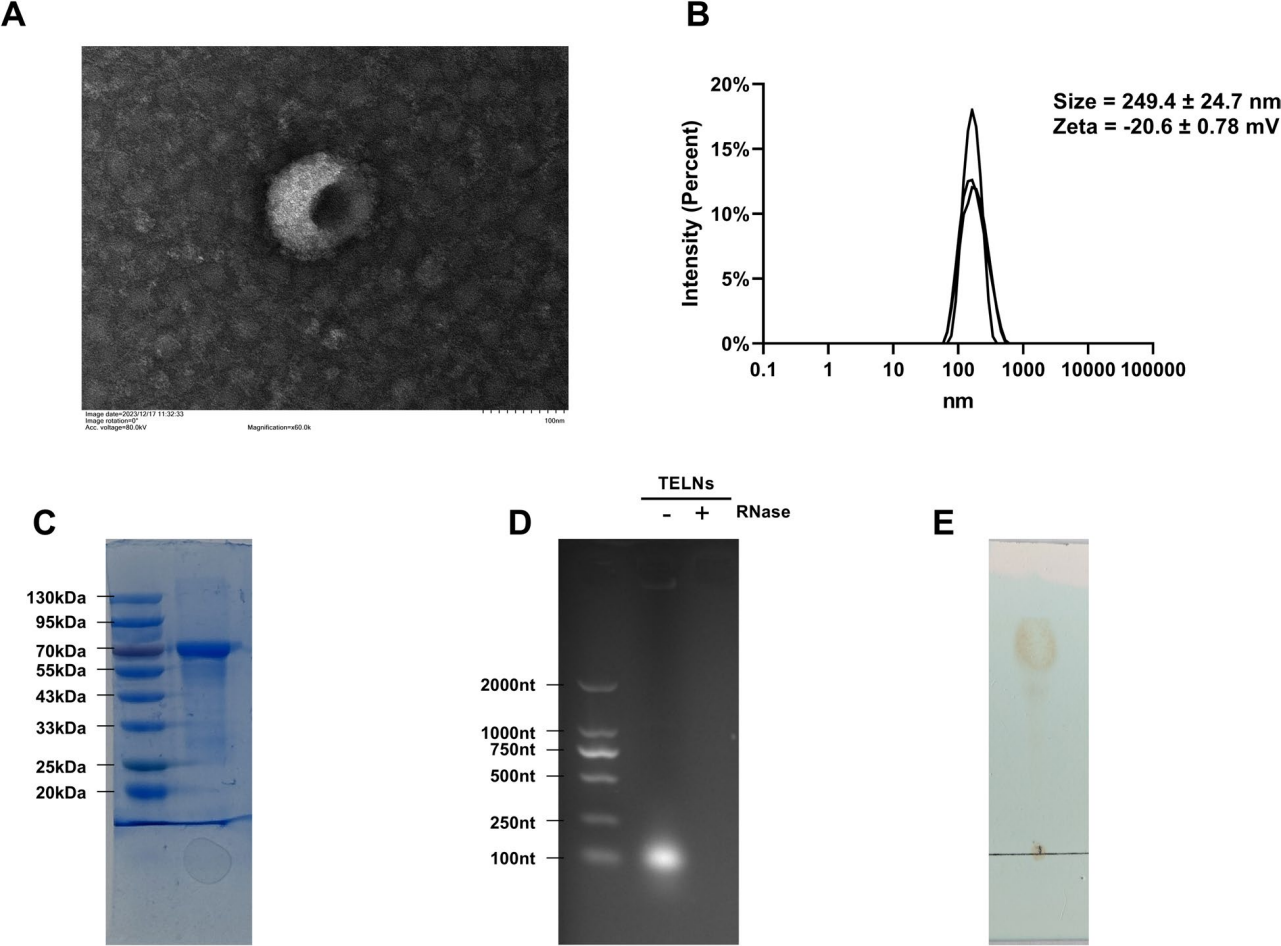
Extraction and characterization of TELNs. **A** TEM. **B** DLS (25 ℃, n = 3). **C** Protein gel. Staining with Coomassie Brilliant Blue. **D** RNA gel. ‘-’ means no RNase was incubated with RNAs and ‘ + ’ means that RNase was incubated with RNase for 30 min at 37 ℃. **E** TLC gel. (full uncropped Gels and Blots images are shown in Supplementary Fig. 1)

A combination of PEG precipitation and ultracentrifugation was employed to extract PELNs from tea leaves. Transmission electron microscopy (TEM) was used to examine the morphology of the PELNs, which revealed a distinct concave spherical structure (Fig. 1A). In addition, dynamic light scattering (DLS) was employed to characterize the particle size and Zeta potential of the PELNs. The results showed that the TELNs had an average particle size of approximately 249 ± 24.7 nm and the Zeta potential of −20.6 ± 0.78 mV, indicating a certain degree of stability in aqueous solution (Fig. 1B). Furthermore, we characterized the contents of the TELNs, which were found to contain proteins, lipid and RNA, consistent with the characteristics of plant-derived exosome-like extracellular vesicles (Fig. 1C–E).

PELNs are considered to have significant potential for application due to their safety, scalability, and cost-effectiveness (Zhang et al. 2016a). Nevertheless, ultracentrifugation, often regarded as the standard method for extracting PELNs, has several limitations, such as hazardous separation processes, low yield per run, and potential damage to PELNs caused by multiple centrifugation cycles (Chen et al. 2022).

In this experiment, we combined PEG precipitation with ultracentrifugation to extract TELNs. Initially, PEG6000 was used for the coarse separation of PELNs. The products from multiple extractions were then collected and subjected to a unified ultracentrifugation process. This strategy enhanced separation efficiency while reducing the frequency of ultracentrifugation steps, thereby minimizing potential damage to PELNs. Additionally, ultracentrifugation effectively removed impurities had that precipitated with PELNs during PEG6000 treatment, ensuring the purity of TELNs. Based on the characterization results, this method successfully isolated TELNs compared to other approaches reported in the literature.

### TELNs were significantly engulfed by HepG-2 cells, with no cytotoxicity detected

The internalization of PELNs by target cells is a critical factor in determining their therapeutic potential. To investigate the uptake of TELNs by HepG-2 cells, the TELNs were labeled with the fluorescent dye PKH26 and isolated using ultracentrifugation. The labeled TELNs were then co-incubated with HepG-2 cells. After 24 h, the cell nuclei were stained with DAPI, and the fluorescence distribution was analyzed using a fluorescence microscope.

**Fig. 2:**
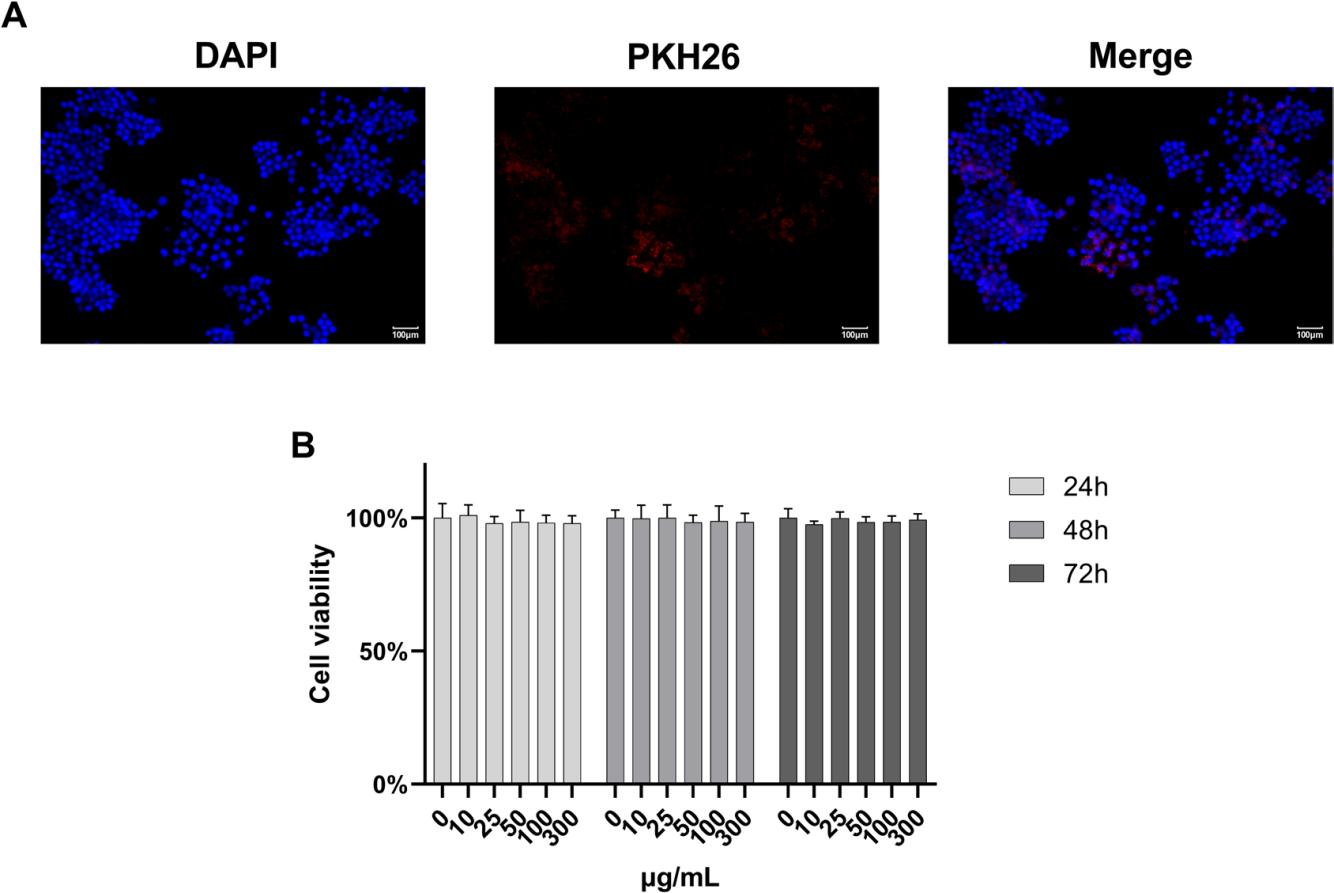
Cellular Uptake and Cytotoxicity of TELNs. **A** Confocal fluorescence microscopy images (200 × , scale bar = 100 μm). **B** Cell viability at 24 h, 48 h, 72 h (n = 5)

The PKH26-labeled TELNs exhibited red fluorescence, while the DAPI-stained nuclei showed blue fluorescence. After 24 h, confocal fluorescence microscopy images (Fig. 2A) demonstrated the presence of red fluorescence within the cells. The merged images revealed that the red fluorescence was closely associated with and surrounded the blue fluorescence of the nuclei, confirming that HepG-2 cells had internalized the TELNs.

We also assessed the cytotoxicity of TELNs. HepG-2 cells were co-incubated with various concentrations of TELNs for 24, 48, and 72 h, and cell viability was measured using the CCK-8 assay (Fig. 2B). The results indicated that even after 72 h of co-incubation at the highest concentration (300 μg/ml), TELNs did not exhibit significant toxicity, nor did they promote cell proliferation. These findings suggest that TELNs, similar to most ELNs derived from edible plants, offer the advantage of low toxicity.

The ability of TELNs to be internalized by HepG-2 cells may be due to their phospholipid bilayer shell. Thin-layer chromatography (TLC) analysis has confirmed that TELNs contain lipid components. Previous research has identified that TELNs include lipids such as phosphatidic acid, phosphatidylglycerol, and phosphatidylcholine (Zu et al. 2021). These lipid components likely contribute to the stability of TELNs and may also facilitate their cellular uptake.

In contrast to previous studies (Chen et al. 2023), our TELNs exhibited no toxicity to HepG-2 cells. We surmise that the main possible causes are presented as follows. Firstly, TELNs do not exhibit high specificity for liver cancer cells. In addition, differences in tea types and extraction methods may also have a potential impact on the biological activity of TELNs.

### TELNs exhibited significant in vitro antioxidant activity

Several studies have indicated that hyperlipidemia and Non-alcoholic Fatty Liver Disease (NAFLD) are often closely linked to oxidative damage in liver cells, and the regular consumption of foods rich in antioxidants can slow the progression of hyperlipidemia and NAFLD (Zhou et al. 2024; Chen et al. 2020; Rahmouni et al. 2019). Therefore, we assessed the antioxidant activity of TELNs.

**Fig. 3:**
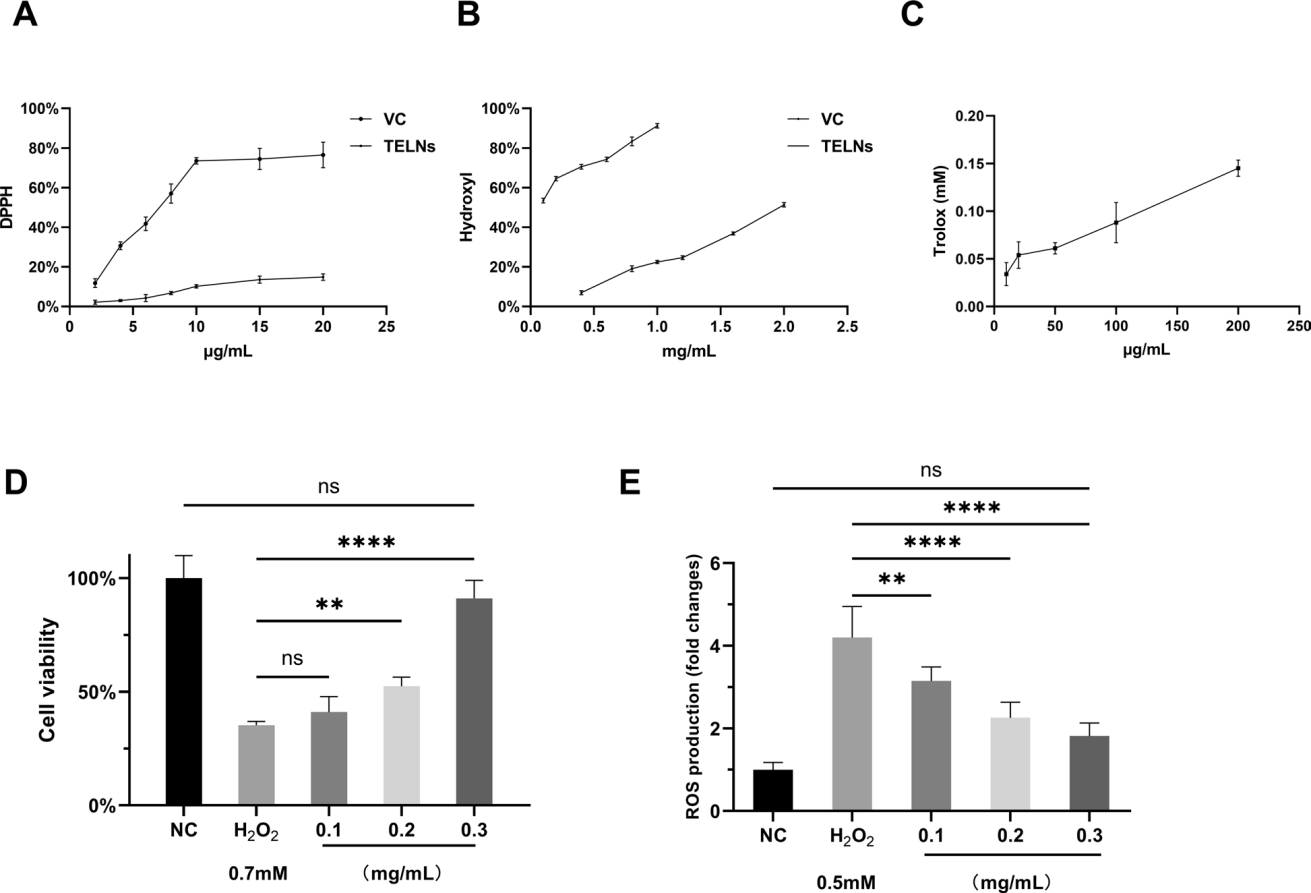
In vitro antioxidant activity of TELNs. **A** DPPH radical scavenging rate. **B** Hydroxyl radical scavenging rate. **C** ABTS radical scavenging rate, expressed as Trolox equivalents (n = 5). **D** Intracellular capacity to counteract oxidative stress (n = 6). **E** Intracellular ROS content (n = 6)

As shown in Fig. 3A–C, the DPPH radical scavenging rate and hydroxyl radical scavenging rate of TELNs were significantly lower than those observed in the control group (Vc). For ABTS radical scavenging, 200 μg/mL of TELNs was equivalent to only 0.145 mM of Trolox, indicating that TELNs exhibit relatively weak in vitro antioxidant activity.

However, it is noteworthy that TELNs effectively mitigated H_2_O_2_-induced oxidative damage in HepG-2 cells. We induced oxidative damage in HepG-2 cells using 0.7 mM H_2_O_2_, resulting in decreasing cell viability. Following co-incubation with varying concentrations of TELNs, the results (Fig. 3D) showed that even at a concentration of 0.2 mg/mL, TELNs significantly alleviated H_2_O_2_-induced oxidative damage, enhancing cell viability (*P* < *0.001*). A dose-dependent effect was observed, with 0.3 mg/mL of TELNs nearly completely reversing the oxidative damage caused by H_2_O_2_ (high-dose group vs. model group: *P* < *0.0001*, high-dose group vs. normal group: *P* > *0.05*).

Reactive oxygen species (ROS) are an inevitable byproduct of cellular metabolism. However, an excessive accumulation of ROS within cells can lead to oxidative stress, which in turn contributes to chronic tissue damage (Xie et al. 2024). Therefore, the level of ROS in cells serves as an important indicator of cellular oxidative stress. In this study, we investigated the changes in ROS levels in HepG-2 cells. The results showed that TELNs significantly reduced the ROS levels induced by H₂O₂. A dose-dependent effect was also observed, with the ROS levels in the 0.3 mg/mL TELNs group showing no significant difference compared to the NC group.

Previous studies have indicated that tea leaves are rich in antioxidants, including catechins, epicatechins, epigallocatechin, and epigallocatechin-3-gallate (Deo et al. 2024). TELNs may contain some of these antioxidant substances (Chen et al. 2023). These substances may be the source of the antioxidant capacity in TELNs (Akacha et al. 2022). In addition, some studies have suggested that the biological activity of PELNs may be related to lipids and miRNA (Zhu et al. 2023; Egea-Jimenez and Zimmermann 2020). Therefore, the biological activity of TELNs may be the result of their combined action.

### TELNs were obviously able to modulating lipid metabolism in HepG-2 cells

**Fig. 4:**
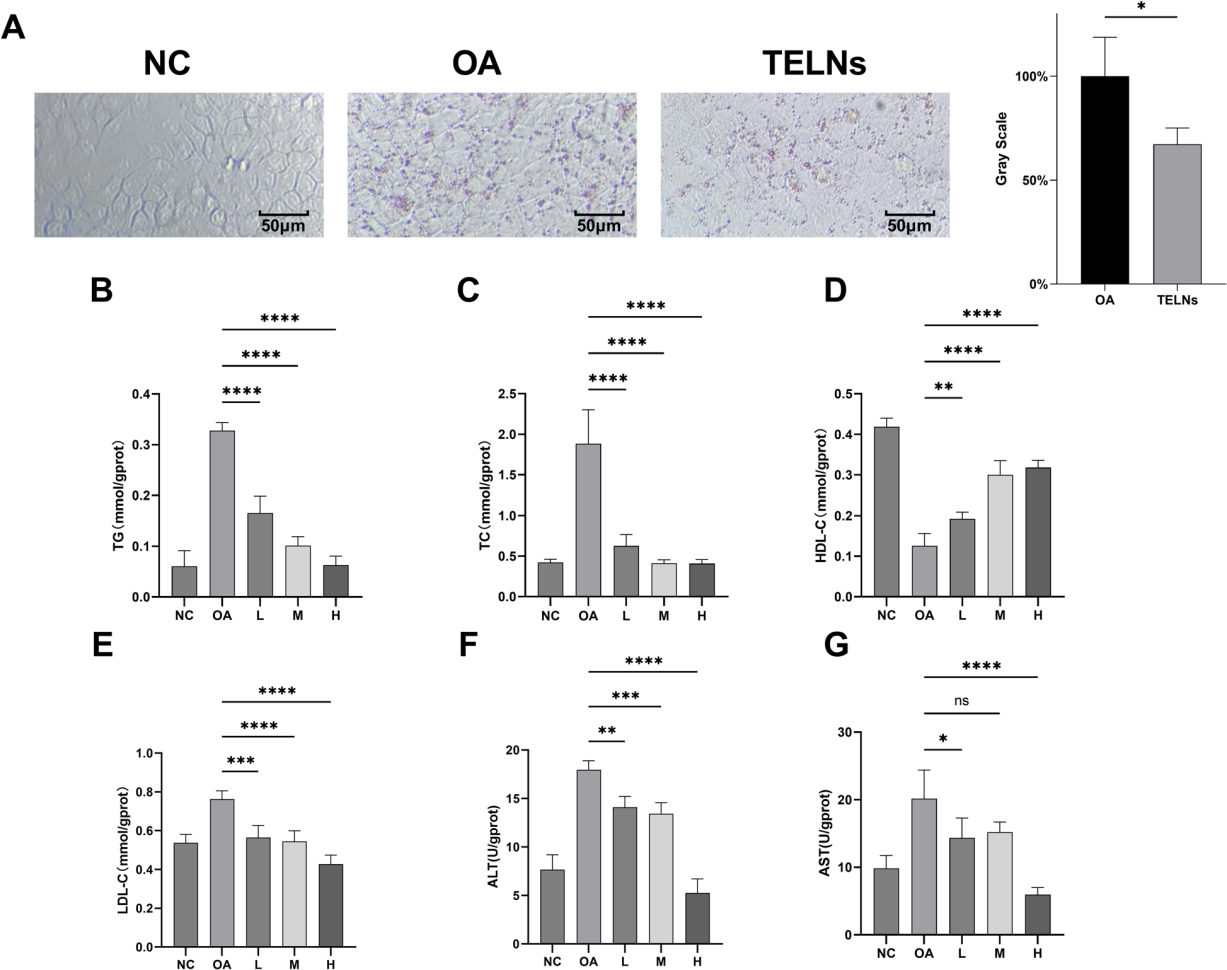
TELNs regulate lipid metabolism in HepG-2 cells. **A** Oil Red O staining and grayscale analysis, TELNs = 300 μg/mL (n = 3). **B**–**G** TG, TC, HDL-C, LDL-C, ALT, and AST measurements. L = 100 μg/mL TELNs, M = 200 μg/mL TELNs, H = 300 μg/mL TELNs (n = 6)

As show in Fig. 4A, The NC group exhibited an absence of red oil droplets, while the OA group displayed extensive red lipid droplets around the cells, indicating significant lipid accumulation induced by OA. This effect was significantly mitigated by TELNs, with a marked reduction in lipid droplets observed in the TELNs group compared to the OA group, as confirmed by relative grayscale analysis (P < 0.05). This suggests that TELNs can effectively reduce OA-induced lipid accumulation.

We further analyzed the levels of triglycerides (TG), total cholesterol (TC), high-density lipoprotein cholesterol (HDL-C), and low-density lipoprotein cholesterol (LDL-C) in HepG-2 cells (Fig. 4B–E). The OA group showed a significant increase in TG, TC, and LDL-C levels (*P* < *0.0001*), accompanied by a significant decrease in HDL-C, compared to the NC group. This indicates that OA co-incubation disrupted lipid metabolism, leading to lipid accumulation in HepG-2 cells. However, TELNs were able to significantly reduce or even reverse these effects. After 24 h of co-incubation with OA and TELNs, the TELNs group exhibited significantly lower TG and TC levels (*P* < *0.0001*), higher HDL-C levels (*P* < *0.01*), and reduced LDL-C levels (*P* < *0.001*) compared to the OA group. These effects were dose-dependent, with increased TELNs concentrations enhancing the outcomes.

ALT and AST are key markers released during liver cell injury and are widely used to assess liver cell damage (Chinnappan et al. 2023). Studies have shown that lipid accumulation in the liver due to disrupted lipid metabolism often leads to liver cell injury. In this study, the OA group exhibited significantly higher ALT (*P* < *0.01*) and AST (*P* < *0.05*) levels compared to the NC group, indicating HepG-2 cell damage under OA treatment (Fig. 4F, G). Notably, TELNs significantly alleviated OA-induced damage, as evidenced by the marked reduction in ALT and AST levels in the TELNs group (*P* < *0.01*).

### TELNs markedly regulated lipid metabolism-related genes

Peroxisome proliferator-activated receptor alpha (PPAR-α), a member of the nuclear hormone receptor superfamily, is activated by a range of lipid ligands (Pawlak et al. 2015). It is intimately involved in hepatic lipid metabolism and serves as the principal regulator of fatty acid β-oxidation, a pivotal process in fat metabolism. Disruption of β-oxidation can lead to the accumulation of excess fatty acids as triglycerides, potentially resulting in NAFLD (Tahri-Joutey et al. 2021). Cholesterol 7α-hydroxylase (CYP7A1), a liver-specific enzyme within the microsomal cytochrome P450 family, acts as the rate-limiting enzyme in the conversion of cholesterol to bile acids in the liver. It is instrumental in the regulation of bile acid metabolism and crucial for the maintenance of cholesterol homeostasis (Chiang and Ferrell 2020). Carnitine palmitoyltransferase 1A (CPT1A), another key enzyme in the β-oxidation of fatty acids, facilitates the transfer of the acyl group from long-chain fatty acyl-CoA to carnitine, marking a critical step in mitochondrial fatty acid β-oxidation.

**Fig. 5:**
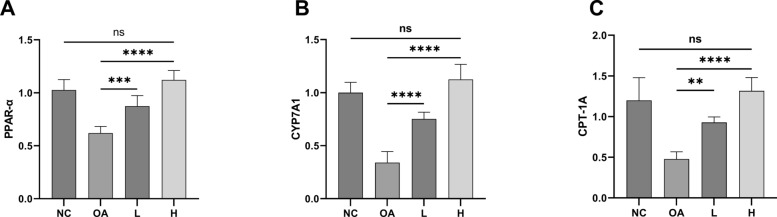
Regulation of lipid metabolism genes by TELNs. **A** PPAR-α. **B** CYP7A1. **C** CPT-1A (n = 6). L = 100 μg/mL TELNs, H = 300 μg/mL TELNs

We assessed the expression levels of the genes mentioned above using RT-qPCR (Fig. 5A–C). In the OA group, gene expression levels of PPAR-α, CYP7A1, and CPT-1A were significantly reduced, indicating that OA adversely affects cholesterol homeostasis and fatty acid β-oxidation in HepG-2 cells. In contrast, the TELNs-treated groups (L and H) showed a significant reversal of this downregulation, with a dose-dependent effect observed. Specifically, in the L group, the expression levels of PPAR-α, CYP7A1, and CPT-1A were significantly higher compared to the OA group (*P* < *0.01*). In the H group, these gene expression levels not only increased further compared to the L group but also nearly reached the levels seen in the normal control group (H-NC, *P* > *0.05*). This suggests that TELNs can fully reverse the suppression of lipid metabolism regulatory genes caused by OA, implying that TELNs can restore normal fatty acid β-oxidation pathways and correct OA-induced lipid metabolism dysregulation and lipid accumulation by improving the expression of these key genes.

### TELNs was capable of significantly regulating lipid metabolism gene expression via miRNA

miRNAs are non-coding endogenous RNA molecules composed of 21–23 nucleotides that regulate gene expression at the post-transcriptional level through the cellular RNA interference pathway(Moraes et al. 2021), and are recognized as significant epigenetic factors influencing the development of NAFLD (Changez et al. 2023). Prior research has demonstrated that patients with NAFLD exhibit dysregulated levels of miRNAs involved in lipid metabolism, inflammation activation, fibrosis, insulin resistance, oxidative stress, and apoptosis. This has given rise to a growing interest in developing miRNA-targeted therapies for NAFLD, although these drugs often come with severe side effects (Gjorgjieva et al. 2019). Therefore, selecting miRNA-targeting compounds from naturally occurring substances with high safety profiles is considered a more prudent approach (Changez et al. 2023). miR-21-5p has been identified as a key player in the development of NAFLD and NASH and is regarded as a significant diagnostic marker for NAFLD (Lin et al. 2022; Rodrigues et al. 2023). miR-21-5p downregulates the expression of PPAR-α, and silencing miR-21-5p can restore PPAR-α expression, thus normalizing lipid metabolism (Goujon et al. 2022). miR-17-3p, a member of the miR-17–92 family, is generally recognized as an oncogene. However, miR-17-3p contributes to lipid accumulation in liver cancer cells by inhibiting the target gene CYP7A1, which is implicated in NAFLD development (Gong et al. 2018). miR-107 has also been shown to inhibit CPT-1A, resulting in lipid accumulation in liver cells (Koushki et al. 2020).

To further elucidate the mechanism through which TELNs regulate lipid metabolism gene expression, we investigated the levels of several miRNAs (miR-21-5p, miR-17-3p, miR-107) in HepG-2 cells.

**Fig. 6:**
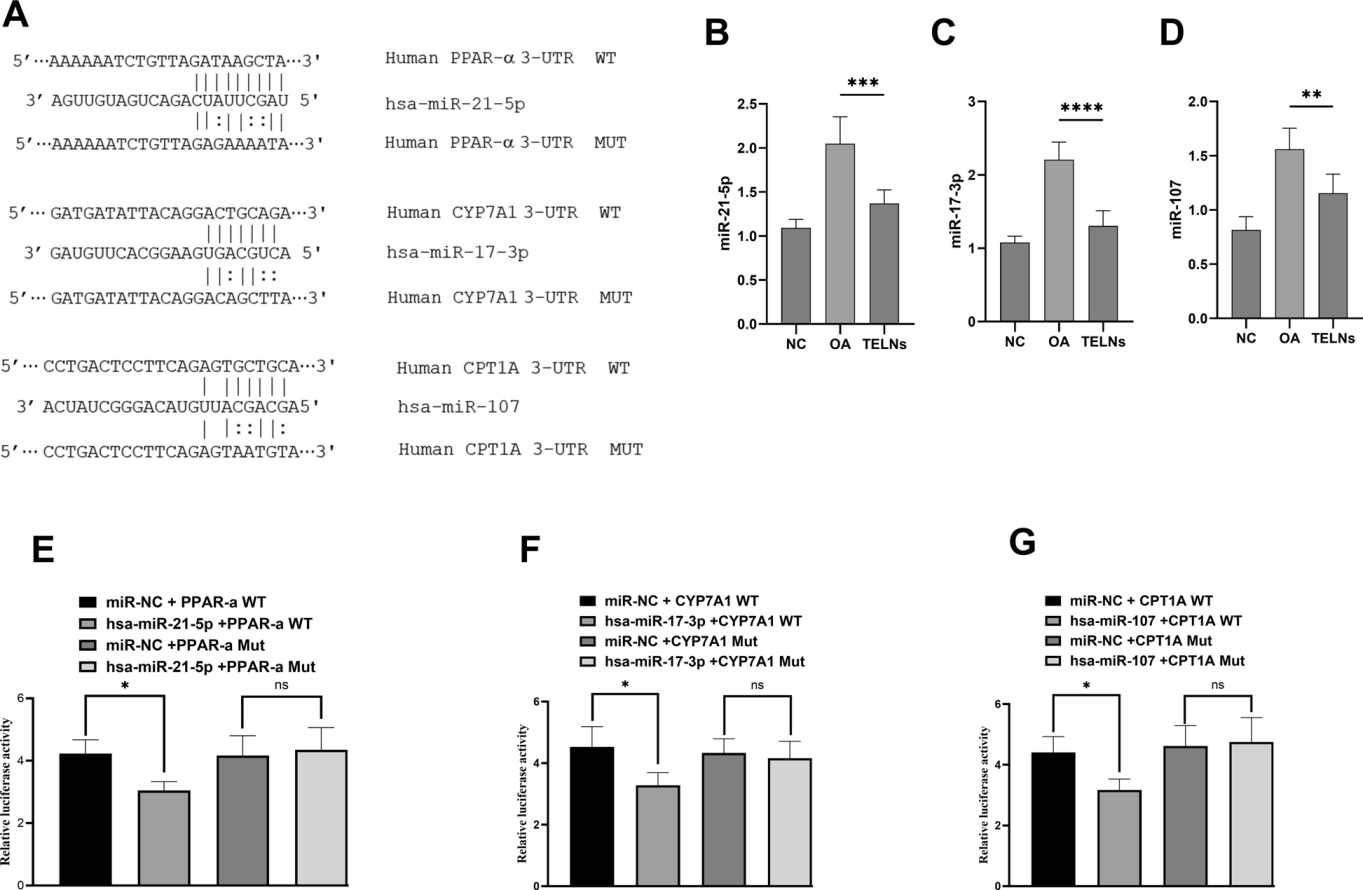
TELNs regulate lipid metabolism gene expression via miRNA. **A** Potential interaction sites. **B**–**D** miR-21-5p, miR-17-3p, and miR-107 expression levels in HepG-2 cells (TELNs = 300 μg/mL, n = 6). **E**, **F** The relative fluorescence values after transfection and mutation indicate that miR-21-5p, miR-17-3p, and miR-107 each exhibit significant interactions with the target genes PPAR-α, CYP7A1, and CPT-1A (n = 6)

The expression levels of the three miRNAs across different groups are shown in Fig. 6A–C. In the OA group, the levels of all three miRNAs were significantly increased compared to the NC group (*P* < *0.01*). Conversely, in the TELNs groups, the expression levels of these miRNAs were significantly reduced compared to the OA group (*P* < *0.01*). Using TargetScan, we predicted potential interaction sites between miR-21-5p, miR-17-3p, miR-107, and PPAR-α, CYP7A1, and CPT1A (Fig. 6D). We further validated these interactions by constructing reporter plasmids with the potential binding sites for the three miRNAs and their corresponding target genes, and performed dual-luciferase reporter assays. The results demonstrated that miR-21-5p, miR-17-3p, and miR-107 significantly reduced the levels of PPAR-α, CYP7A1, and CPT1A wild-type constructs in 293T cells (*P* < *0.05*) (Fig. 6E, F).

Previous experiments have demonstrated that TELNs can be taken up by HepG-2 cells, and when TELNs are taken up by HepG-2 cells, the cargoes (lipids, proteins, RNAs, biologically active substances) carried therein likewise enter the cells and produce the corresponding bioactivities thus indirectly affecting the miRNA content in the cells. In contrast, Yan et al. found that *Brucea javanica* derived exosome-like nanovesicles carries some of the miRNAs that are also found in animal cells, and thus when it enters the cell, it is able to directly regulate the miRNA content(Yan et al. 2024). We suggest that TELNs may have regulated miRNA levels in HepG-2 cells through both pathways.

Therefore, TELNs may mitigate the inhibitory effects of the elevated miR-21-5p, miR-17-3p, and miR-107 levels induced by OA on the expression of PPAR-α, CYP7A1, and CPT1A. By reducing the levels of these miRNAs, TELNs enhance the expression of PPAR-α, CYP7A1, and CPT1A, leading to improved lipid metabolism in HepG-2 cells.

In this study, we characterized TELNs in terms of both morphology and composition. However, we did not investigate some marker proteins present in Exosomes due to the lack of recognized marker proteins for the characterization of PELNs (Pinedo et al. 2021). Second, we demonstrated that TELNs can be taken up by HepG-2 cells; however, the exact process and channels remain unproven. Lastly, as TELNs may need to exist within the gastrointestinal environment of animals in future studies, research on the gastrointestinal stability of TELNs is imperative. It is hoped that the enhancement of experimental methodologies and the profundity of research will facilitate the remediation of the aforementioned deficiencies.

In summary, TELNs were successfully isolated using a combination of PEG precipitation and ultracentrifugation techniques. These TELNs exhibited an average particle size of approximately 249 ± 24.7 nm and a Zeta potential of -20.6 ± 0.78 mV. They are readily internalized by HepG-2 cells without inducing cytotoxicity. Experimental data showed that TELNs effectively ameliorated OA-induced disruption of lipid metabolism and hepatocyte injury by down-regulating the expression of miR-21-5p, miR-17-3p, and miR-107 and leading to the subsequent up-regulation of their target genes in HepG-2 cells, thereby ameliorating the process of lipid metabolism affected by OA.

## Supplementary Information

Supplementary material 1.

## Data Availability

The datasets used and/or analyzed during the current study are available from the corresponding author on reasonable request.

## Abbreviations

TELNs: Tea leaf exosome-like nanoparticles
PELNs: Plant-derived exosome-like nanoparticles
PEG: Polyethylene glycol
OA: Oleic acid
DLS: Dynamic light scattering
DMEM: Dulbecco's modified eagle medium
FBS: Fetal bovine serum
TG: Triglycerides
TC: Total cholesterol
LDL-C: Low density lipoprotein cholesterol
HDL-C: High density lipoprotein cholesterol
AST: Aspartate aminotransferase
ALT: Alanine aminotransferase
Vc: Vitamin C
TEM: Transmission electron microscopy
TLC: Thin-layer chromatography
PPAR-α: Peroxisome proliferator-activated receptor alpha
CYP7A1: Cholesterol 7α-hydroxylase
CPT1A: Carnitine palmitoyltransferase 1A
NAFLD: Non-alcoholic fatty liver disease
