# Supplementary material for: Tea leaf exosome-like nanoparticles (TELNs) improve oleic acid-induced lipid metabolism by regulating miRNAs in HepG-2 cells
Source: Bioresour Bioprocess. 2025 Feb 10;12(1):9. doi: 10.1186/s40643-025-00844-1 (PMC11810870; doi:10.1186/s40643-025-00844-1)
Supplement: Supplementary file 1 — Supplementary material 1. [file 40643_2025_844_MOESM1_ESM.docx]

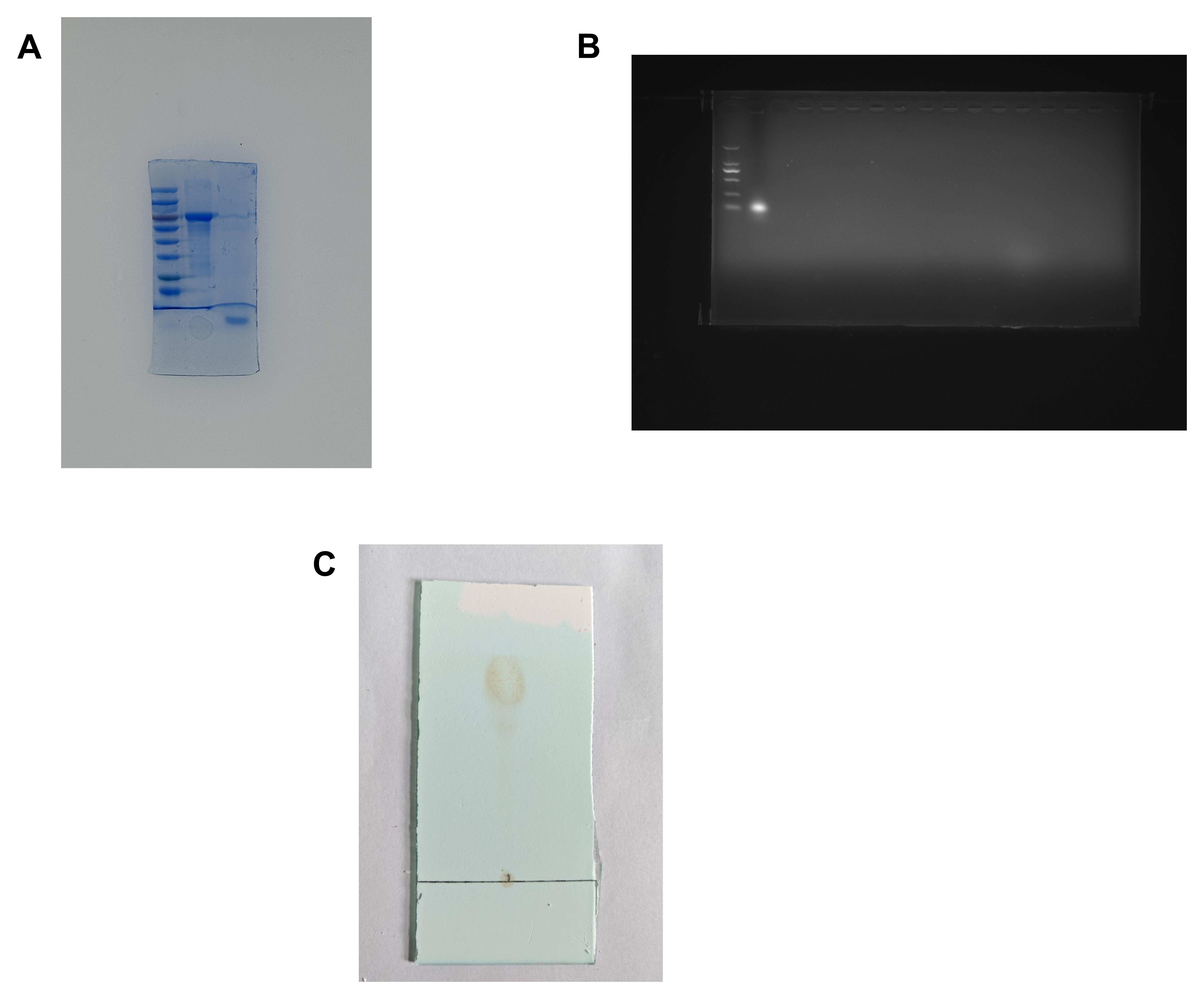


**Supplementary Figure 1** full uncropped Gels and Blots images. A) SDS-PAGE electrophoresis with Coomassie Brilliant Blue staining. B) 1.5% agarose gel electrophoresis. C) thin-layer chromatography.
